# Hand Gesture Recognition Based on Computer Vision: A Review of Techniques

**DOI:** 10.3390/jimaging6080073

**Published:** 2020-07-23

**Authors:** Munir Oudah, Ali Al-Naji, Javaan Chahl

**Affiliations:** 1 Electrical Engineering Technical College, Middle Technical University, Baghdad 10022, Iraq; Munir_aliraqi@yahoo.com; 2 School of Engineering, University of South Australia, Mawson Lakes SA 5095, Australia; Javaan.Chahl@unisa.edu.au

**Keywords:** hand gesture, hand posture, computer vision, human–computer interaction (HCI)

## Abstract

Hand gestures are a form of nonverbal communication that can be used in several fields such as communication between deaf-mute people, robot control, human–computer interaction (HCI), home automation and medical applications. Research papers based on hand gestures have adopted many different techniques, including those based on instrumented sensor technology and computer vision. In other words, the hand sign can be classified under many headings, such as posture and gesture, as well as dynamic and static, or a hybrid of the two. This paper focuses on a review of the literature on hand gesture techniques and introduces their merits and limitations under different circumstances. In addition, it tabulates the performance of these methods, focusing on computer vision techniques that deal with the similarity and difference points, technique of hand segmentation used, classification algorithms and drawbacks, number and types of gestures, dataset used, detection range (distance) and type of camera used. This paper is a thorough general overview of hand gesture methods with a brief discussion of some possible applications.

## 1. Introduction

Hand gestures are an aspect of body language that can be conveyed through the center of the palm, the finger position and the shape constructed by the hand. Hand gestures can be classified into static and dynamic. As its name implies, the static gesture refers to the stable shape of the hand, whereas the dynamic gesture comprises a series of hand movements such as waving. There are a variety of hand movements within a gesture; for example, a handshake varies from one person to another and changes according to time and place. The main difference between posture and gesture is that posture focuses more on the shape of the hand whereas gesture focuses on the hand movement. The main approaches to hand gesture research can be classified into the wearable glove-based sensor approach and the camera vision-based sensor approach [1,2].

Hand gestures offer an inspiring field of research because they can facilitate communication and provide a natural means of interaction that can be used across a variety of applications. Previously, hand gesture recognition was achieved with wearable sensors attached directly to the hand with gloves. These sensors detected a physical response according to hand movements or finger bending. The data collected were then processed using a computer connected to the glove with wire. This system of glove-based sensor could be made portable by using a sensor attached to a microcontroller.

**Figure 1:**
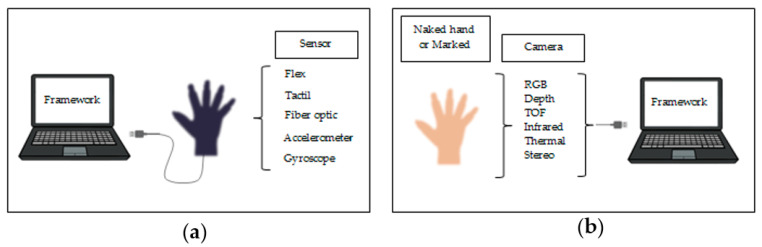
Different techniques for hand gestures. (**a**) Glove-based attached sensor either connected to the computer or portable; (**b**) computer vision–based camera using a marked glove or just a naked hand.

As illustrated in Figure 1, hand gestures for human–computer interaction (HCI) started with the invention of the data glove sensor. It offered simple commands for a computer interface. The gloves used different sensor types to capture hand motion and position by detecting the correct coordinates of the location of the palm and fingers [3]. Various sensors using the same technique based on the angle of bending were the curvature sensor [4], angular displacement sensor [5], optical fiber transducer [6], flex sensors [7] and accelerometer sensor [8]. These sensors exploit different physical principles according to their type.

Although the techniques mentioned above have provided good outcomes, they have various limitations that make them unsuitable for the elderly, who may experience discomfort and confusion due to wire connection problems. In addition, elderly people suffering from chronic disease conditions that result in loss of muscle function may be unable to wear and take off gloves, causing them discomfort and constraining them if used for long periods. These sensors may also cause skin damage, infection or adverse reactions in people with sensitive skin or those suffering burns. Moreover, some sensors are quite expensive. Some of these problems were addressed in a study by Lamberti and Camastra [9], who developed a computer vision system based on colored marked gloves. Although this study did not require the attachment of sensors, it still required colored gloves to be worn.

These drawbacks led to the development of promising and cost-effective techniques that did not require cumbersome gloves to be worn. These techniques are called camera vision-based sensor technologies. With the evolution of open-source software libraries, it is easier than ever to detect hand gestures that can be used under a wide range of applications like clinical operations [10], sign language [11], robot control [12], virtual environments [13], home automation [14], personal computer and tablet [15], gaming [16]. These techniques essentially involve replacement of the instrumented glove with a camera. Different types of camera are used for this purpose, such as RGB camera, time of flight (TOF) camera, thermal cameras or night vision cameras.

Algorithms have been developed based on computer vision methods to detect hands using these different types of cameras. The algorithms attempt to segment and detect hand features such as skin color, appearance, motion, skeleton, depth, 3D model, deep learn detection and more. These methods involve several challenges, which are discussed in this paper in the following sections.

Several studies based on computer vision techniques were published in the past decade. A study by Murthy et al. [17] covered the role and fundamental technique of HCI in terms of the recognition approach, classification and applications, describing computer vision limitations under various conditions. Another study by Khan et al. [18] presented a recognition system concerned with the issue of feature extraction, gesture classification, and considered the application area of the studies. Suriya et al. [19] provided a specific survey on hand gesture recognition for mouse control applications, including methodologies and algorithms used for human–machine interaction. In addition, they provided a brief review of the hidden Markov model (HMM). A study by Sonkusare et al. [20] reported various techniques and made comparisons between them according to hand segmentation methodology, tracking, feature extraction, recognition techniques, and concluded that the recognition rate was a tradeoff with temporal rate limited by computing power. Finally, Kaur et al. [16] reviewed several methods, both sensor-based and vision-based, for hand gesture recognition to improve the precision of algorithms through integrating current techniques.

The studies above give insight into some gesture recognition systems under various scenarios, and address issues such as scene background limitations, illumination conditions, algorithm accuracy for feature extraction, dataset type, classification algorithm used and application. However, no review paper mentions camera type, distance limitations or recognition rate. Therefore, the objective of this study is to provide a comparative review of recent studies concerning computer vision techniques with regard to hand gesture detection and classification supported by different technologies. The current paper discusses the seven most reported approaches to the problem such as skin color, appearance, motion, skeleton, depth, 3D-model, deep-learning. This paper also discusses these approaches in detail and summarizes some modern research under different considerations (type of camera used, resolution of the processed image or video, type of segmentation technique, classification algorithm used, recognition rate, type of region of interest processing, number of gestures, application area, limitation or invariant factor, and detection range achieved and in some cases data set use, runtime speed, hardware run, type of error). In addition, the review presents the most popular applications associated with this topic.

**Figure 2:**
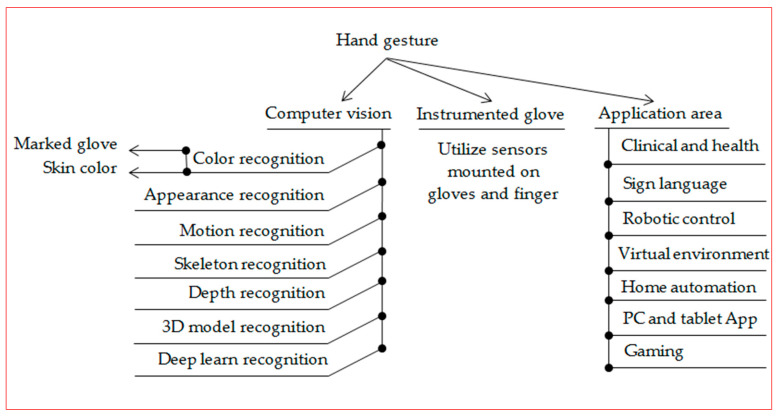
Classifications method conducted by this review.

The remainder of this paper is summarized as follows. Section 2 explains hand gesture methods and take consideration and focus on computer vision techniques, where describe seven most common techniques such as skin color, appearance, motion, skeleton, depth, 3D-module, deep learn and support that with tables. Section 3 illustrates in detail seven application areas that deal with hand gesture recognition systems. Section 4 briefly discusses research gaps and challenges. Finally, Section 5 presents our conclusions. Figure 2 below clarify the classification methods conducted by this review.

## 2. Hand Gesture Methods

The primary goal in studying gesture recognition is to introduce a system that can detect specific human gestures and use them to convey information or for command and control purposes. Therefore, it includes not only tracking of human movement, but also the interpretation of that movement as significant commands. Two approaches are generally used to interpret gestures for HCI applications. The first approach is based on data gloves (wearable or direct contact) and the second approach is based on computer vision without the need to wear any sensors.

### 2.1. Hand Gestures Based on Instrumented Glove Approach

**Figure 3:**
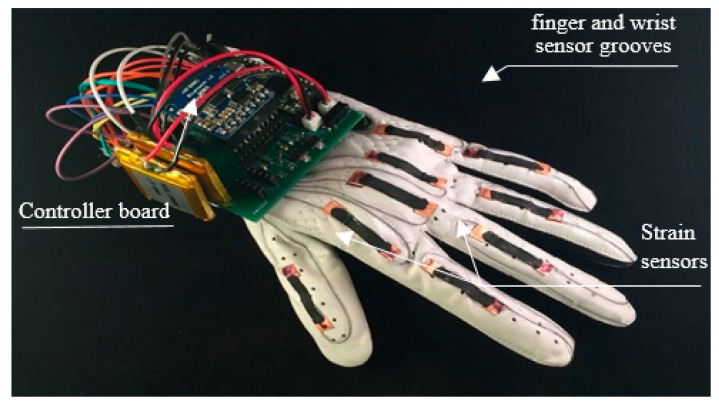
Sensor-based data glove (adapted from website: https://physicsworld.com/a/smart-glove-translates-sign-language-into-digital-text/).

The wearable glove-based sensors can be used to capture hand motion and position. In addition, they can easily provide the exact coordinates of palm and finger locations, orientation and configurations by using sensors attached to the gloves [21,22,23]. However, this approach requires the user to be connected to the computer physically [23], which blocks the ease of interaction between user and computer. In addition, the price of these devices is quite high [23,24]. However, the modern glove based approach uses the technology of touch, which more promising technology and it is considered Industrial-grade haptic technology. Where the glove gives haptic feedback that makes user sense the shape, texture, movement and weight of a virtual object by using microfluidic technology. Figure 3 shows an example of a sensor glove used in sign language.

### 2.2. Hand Gestures Based on Computer Vision Approach

**Figure 4:**
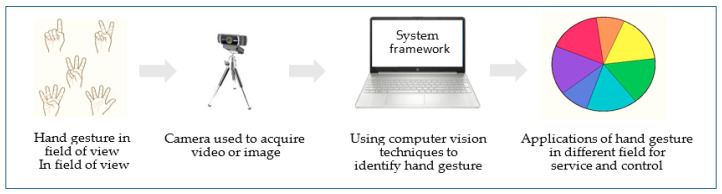
Using computer vision techniques to identify gestures. Where the user perform specific gesture by single or both hand in front of camera which connect with system framework that involve different possible techniques to extract feature and classify hand gesture to be able control some possible application.

The camera vision based sensor is a common, suitable and applicable technique because it provides contactless communication between humans and computers [16]. Different configurations of cameras can be utilized, such as monocular, fisheye, TOF and IR [20]. However, this technique involves several challenges, including lighting variation, background issues, the effect of occlusions, complex background, processing time traded against resolution and frame rate and foreground or background objects presenting the same skin color tone or otherwise appearing as hands [17,21]. These challenges will be discussed in the following sections. A simple diagram of the camera vision-based sensor for extracting and identifying hand gestures is presented in Figure 4.

#### 2.2.1. Color-Based Recognition:

##### Color-Based Recognition Using Glove Marker

**Figure 5:**
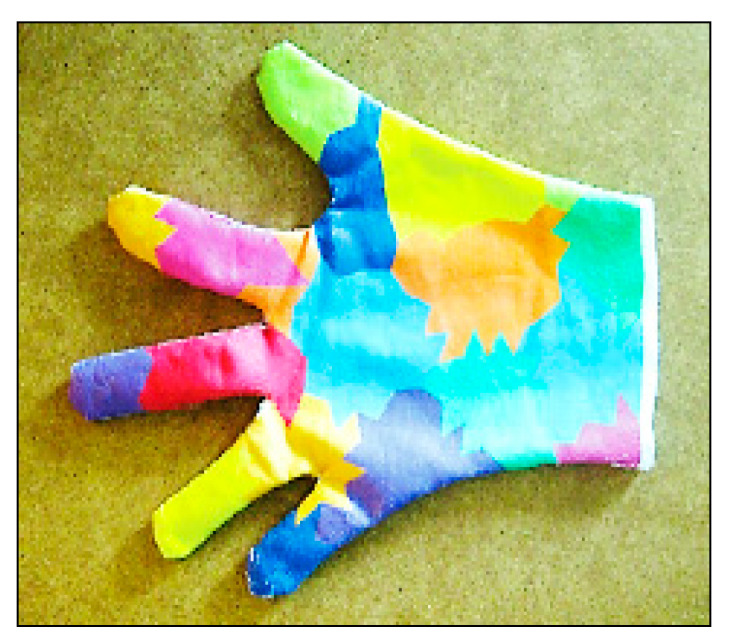
Color-based recognition using glove marker [13].

This method uses a camera to track the movement of the hand using a glove with different color marks, as shown in Figure 4. This method has been used for interaction with 3D models, permitting some processing, such as zooming, moving, drawing and writing using a virtual keyboard with good flexibility [9]. The colors on the glove enable the camera sensor to track and detect the location of the palm and fingers, which allows for the extraction of geometric model of the shape of the hand [13,25]. The advantages of this method are its simplicity of use and low price compared with the sensor data glove [9]. However, it still requires the wearing of colored gloves and limits the degree of natural and spontaneous interaction with the HCI [25]. The color-based glove marker is shown in Figure 5 [13].

##### Color-Based Recognition of Skin Color

Skin color detection is one of the most popular methods for hand segmentation and is used in a wide range of applications, such as object classification, degraded photograph recovery, person movement tracking, video observation, HCI applications, facial recognition, hand segmentation and gesture identification. Skin color detection has been achieved using two methods. The first method is pixel based skin detection, in which each pixel in an image is classified into skin or not, individually from its neighbor. The second method is region skin detection, in which the skin pixels are spatially processed based on information such as intensity and texture.

**Figure 6:**
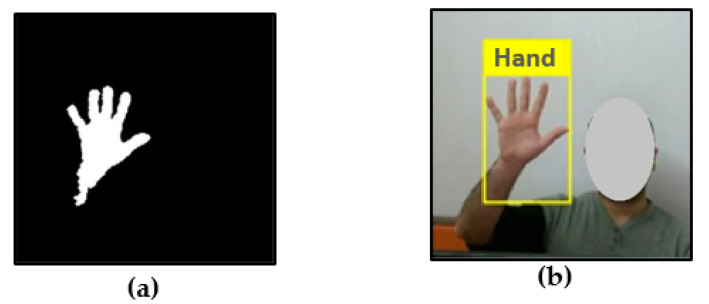
Example of skin color detection. (**a**) Apply threshold to the channels of YUV color space in order to extract only skin color then assign 1 value for the skin and 0 to non-skin color; (**b**) detected and tracked hand using resulted binary image.

Color space can be used as a mathematical model to represent image color information. Several color spaces can be used according to the application type such as digital graphics, image process applications, TV transmission and application of computer vision techniques [26,27]. Figure 6 shows an example of skin color detection using YUV color space.

A several formats of color space are obtained for skin segmentation, as itemized below:

- red, green, blue (R–G–B and RGB-normalized);
- hue and saturation (H–S–V, H–S–I and H–S–L);
- luminance (YIQ, Y–Cb–Cr and YUV).

More detailed discussion of skin color detection based on RGB channels can be found in [28,29]. However, it is not preferred for skin segmentation purposes because the mixture of the color channel and intensity information of an image has irregular characteristics [26]. Skin color can detect the threshold value of three channels (red, green and blue). In the case of normalized-RGB, the color information is simply separated from the luminance. However, under lighting variation, it cannot be relied on for segmentation or detection purposes, as shown in the studies [30,31].

The characteristics of color space such as hue/saturation family and luminance family are good under lighting variations. The transformation of format RGB to HSI or HSV takes time in case of substantial variation in color value (hue and saturation). Therefore, a pixel within a range of intensity is chosen. The RGB to HSV transformation may consume time because of the transformation from Cartesian to polar coordinates. Thus, HSV space is useful for detection in simple images.

Transforming and splitting channels of Y–Cb–Cr color space is simple if compared with the HSV color family in regard to skin color detection and segmentation, as illustrated in [32,33]. Skin tone detection based Y–Cb–Cr is demonstrated in detail in [34,35].

The image is processed to convert RGB color space to another color space in order to detect the region of interest, normally a hand. This method can be used to detect the region through the range of possible colors, such as red, orange, pink and brown. The training sample of skin regions is studied to obtain the likely range of skin pixels with the band values for R, G and B pixels. To detect skin regions, the pixel color should compare the colors in the region with the predetermined sample color. If similar, then the region can be labeled as skin [36]. Table 1 presents a set of research papers that use different techniques to detect skin color.

The skin color method involves various challenges, such as illumination variation, background issues and other types of noise. A study by Perimal et al. [37] provided 14 gestures under controlled-conditions room lighting using an HD camera at short distance (0.15 to 0.20 m) and, the gestures were tested with three parameters, noise, light intensity and size of hand, which directly affect recognition rate. Another study by Sulyman et al. [38] observed that using Y–Cb–Cr color space is beneficial for eliminating illumination effects, although bright light during capture reduces the accuracy. A study by Pansare et al. [11] used RGB to normalize and detect skin and applied a median filter to the red channel to reduce noise on the captured image. The Euclidian distance algorithm was used for feature matching based on a comprehensive dataset. A study by Rajesh et al. [15] used HSI to segment the skin color region under controlled environmental conditions, to enable proper illumination and reduce the error.

Another challenge with the skin color method is that the background must not contain any elements that match skin color. Choudhury et al. [39] suggested a novel hand segmentation based on combining the frame differencing technique and skin color segmentation, which recorded good results, but this method is still sensitive to scenes that contain moving objects in the background, such as moving curtains and waving trees. Stergiopoulou et al. [40] combined motion-based segmentation (a hybrid of image differencing and background subtraction) with skin color and morphology features to obtain a robust result that overcomes illumination and complex background problems. Another study by Khandade et al. [41] used a cross-correlation method to match hand segmentation with a dataset to achieve better recognition. Karabasi et al. [42] proposed hand gestures for deaf-mute communication based on mobile phones, which can translate sign language using HSV color space. Zeng et al. [43] presented a hand gesture method to assist wheelchair users indoors and outdoors using red channel thresholding with a fixed background to overcome the illumination change. A study by Hsieh et al. [44] used face skin detection to define skin color. This system can correctly detect skin pixels under low lighting conditions, and even when the face color is not in the normal range of skin chromaticity. Another study, by Bergh et al. [45], proposed a hybrid method based on a combination of the histogram and a pre-trained Gaussian mixture model to overcome lighting conditions. Pansare et al. [46] aligned two cameras (RGB and TOF) together to improve skin color detection with the help of the depth property of the TOF camera to enhance detection and face background limitations.

#### 2.2.2. Appearance-Based Recognition

This method depends on extracting the image features in order to model visual appearance such as hand and comparing these parameters with feature extracted from the input image frames. Where the features are directly calculated by the pixel intensities without a previous segmentation process. The method is executed in real time due to the easy 2D image features extracted and is considered easier to implement than the 3D model method. In addition, this method can detect various skin tones. Utilizing the AdaBoost learning algorithm, which maintains fixed feature such as key points for a portion of a hand, which can solve the occlusion issue [47,48], it can separate into two models: a motion model and a 2D static model. Table 2 presents a set of research papers that use different segmentation techniques based on appearance recognition to detect region of interest (ROI).

**Figure 7:**
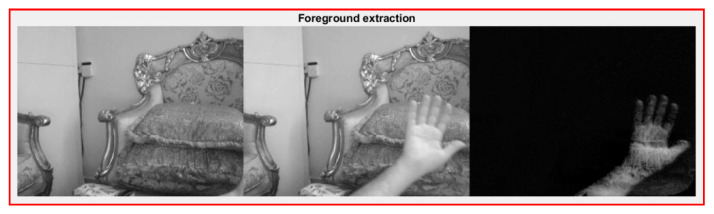
Example on appearance recognition using foreground extraction in order to segment only ROI, where the object features can be extracted using different techniques such as pattern or image subtraction and foreground and background segmentation algorithms.

A study by Chen et al. [49] proposed two approaches for hand recognition. The first approach focused on posture recognition using Haar-like features, which can describe the hand posture pattern effectively used the AdaBoost learning algorithm to speed up the performance and thus rate of classification. The second approach focused on gesture recognition using context-free grammar to analyze the syntactic structure based on the detected postures. Another study by Kulkarni and Lokhande [50] used three feature extraction method such as a histogram technique to segment and observe images that contained a large number of gestures, then suggested using edge detection such as Canny, Sobel and Prewitt operators to detect the edges with a different threshold. The classification gesture performed using feed forward back propagation artificial neural network with supervision learns. Some of the limitation reported by the author where conclude when use histogram technique the system gets misclassified result because histogram can only be used for the small number of gesture which completely different from each other. Fang et al. [51] used an extended AdaBoost method for hand detection and combined optical flow with the color cue for tracking. They also collected hand color from the neighborhood of features’ mean position using a single Gaussian model to describe hand color in HSV color space. Where multi feature extracted and gesture recognition using palm and finger decomposition, then utilizing scale-space feature detection where integrated into gesture recognition in order to encounter the limitation of aspect ratio which facing most of the learning of hand gesture methods. Licsa’r et al. [52] used a simple background subtraction method for hand segmentation and extended it to handle background changes in order to face some challenges such as skin like color and complex and dynamic background then used boundary-based method to classify hand gesture. Finally, Zhou et al. [53] proposed a novel method to directly extract the fingers where the edges were extracted from the gesture images, and then the finger central area was obtained from the obtained edges. Fingers were then obtained from the parallel edge characteristics. The proposed system cannot recognize the side view of hand pose. Figure 7 below show simple example on appearance recognition.

According to information mentioned in Table 2. The first row indicates Haar-like feature which consider a good for analyze ROI pattern efficiently. Haar-like features can efficiently analyze the contrast between dark and bright object within a kernel, which can operate faster compared with pixel based system. In addition, it is immune for noise and lighting variation because they calculate the gray value difference between the white and black rectangles. The result of first row is 90%, but if compared with single gaussian model which used to describe hand color in HSV color space in the third row the result of recognition rate is 93%. Although both proposed system used the Adaboost algorithm to speed up the system and classification.

#### 2.2.3. Motion-Based Recognition

Motion-based recognition can be utilized for detection purposes; it can be extracts the object through a series of image frames. The AdaBoost algorithm utilized for object detection, characterization, movement modeling, and pattern recognition is needed to recognize the gesture [16]. The main issue encounter motion recognition is this is an occasion if one more gesture is active at the recognition process and also dynamic background has a negative effect. In addition, the loss of gesture may be caused by occlusion among tracked hand gesture or error in region extraction from tracked gesture and effect long-distance on the region appearance Table 3 presents a set of research papers that used different segmentation techniques based on motion recognition to detect ROI.

**Figure 8:**
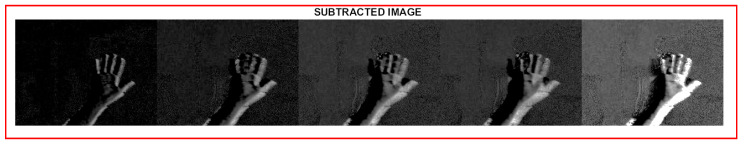
Example on motion recognition using frame difference subtraction to extract hand feature, where the moving object such as hand extracted from the fixed background.

Two stages for efficient hand detection were proposed in [54]. First, the hand detected for each frame and center point is used for tracking the hand. Then, the second stage matching model applying to each type of gesture using a set of features is extracted from the motion tracking in order to provide better classification where the main drawback of the skin color is affected by lighting variations which lead to detect non-skin color. A standard face detection algorithm and optical flow computation was used by [55] to give a user-centric coordinate frame in which motion features were used to recognize gestures for classification purposes using the multiclass boosting algorithm. A real-time dynamic hand gesture recognition system based on TOF was offered in [56], in which motion patterns were detected based on hand gestures received as input depth images. These motion patterns were compared with the hand motion classifications computed from the real dataset videos which do not require the use of a segmentation algorithm. Where the system provides good result except the depth rang limitation of TOF camera. In [57], YUV color space was used, with the help of the CAMShift algorithm, to distinguish between background and skin color, and the naïve Bayes classifier was implemented to assist with gesture recognition. The proposed system faces some challenges such as illumination variation where light changes affect the result of the skin segment. Other challenges are the degree of gesture freedom which affect directly on the output result by change rotation. Next, hand position capture problem, if hand appears in the corner of the frame and the dots which must cover the hand does not lie on hand that may led to failing captured user gesture. In addition, the hand size quite differs between humans and maybe causes a problem with the interaction system. However, the major still challenging problem is the skin-like color which affects overall system and can abort the result. Figure 8 gives simple example on hand motion recognition.

According to information mentioned in Table 3. The first row recognition rate of system is 97%, where the hybrid system based on skin detect and motion detection is more reliable for gesture recognition, where the motion hand can track using multiple track candidates depend on stand derivation calculation for both skin and motion approach. Where every single gesture encoded as chain-code in order to model every single gesture which considers a simple model compared with (HMM) and classified gesture using a model of the histogram distribution. The proposed system in the third row use depth camera based on (TOF) where the motion pattern of the arm model for human utilized to define motion patterns, were the authors confirm that using the depth information for hand trajectories estimation is to improve gesture recognition rate. Moreover, the proposed system no need for the segmentation algorithm, where the system is examined using 2D and 2.5D approaches, were 2.5D performs better than 2D and gives recognition rate 95%.

#### 2.2.4. Skeleton-Based Recognition

The skeleton-based recognition specifies model parameters which can improve the detection of complex features [16]. Where the various representations of skeleton data for the hand model can be used for classification, it describes geometric attributes and constraint and easy translates features and correlations of data, in order to focus on geometric and statistic features. The most common feature used is the joint orientation, the space between joints, the skeletal joint location and degree of angle between joints and trajectories and curvature of the joints. Table 4 presents a set of research papers that use different segmentation techniques based on skeletal recognition to detect ROI.

**Figure 9:**
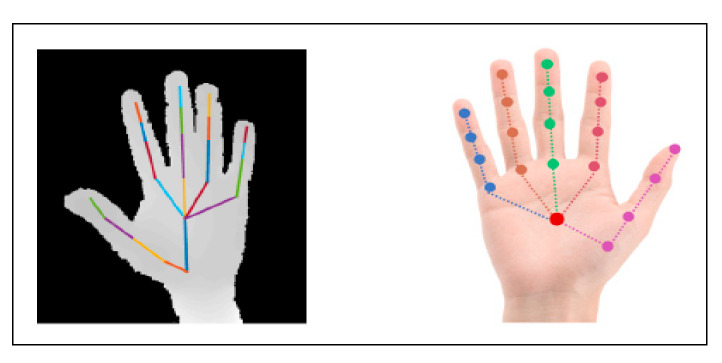
Example of skeleton recognition using depth and skeleton dataset to representation hand skeleton model [62].

Hand segmentation using the depth sensor of the Kinect camera, followed by location of the fingertips using 3D connections, Euclidean distance, and geodesic distance over hand skeleton pixels to provide increased accuracy was proposed in [58]. A new 3D hand gesture recognition approach based on a deep learning model using parallel convolutional neural networks (CNN) to process hand skeleton joints’ positions was introduced in [59], the proposed system has a limitation where it works only with complete sequence. The optimal viewpoint was estimated and the point cloud of gesture transformed using a curve skeleton to specify topology, then Laplacian-based contraction was applied to specify the skeleton points in [60]. Where the Hungarian algorithm was applied to calculate the match scores of the skeleton point set, but the joint tracking information acquired by Kinect is not accurate enough which give a result with constant vibration. A novel method based on skeletal features extracted from RGB recorded video of sign language, which presents difficulties to extracting accurate skeletal data because of occlusions, was offered in [61]. A dynamic hand gesture using depth and skeletal dataset for a skeleton-based approach was presented in [62], where supervised learning (SVM) used for classification with a linear kernel. Another dynamic hand gesture recognition using Kinect sensor depth metadata for acquisition and segmentation which used to extract orientation feature, where the support vector machine (SVM) algorithm and HMM was utilized for classification and recognition to evaluate system performance where the SVM bring a good result than HMM in some specification such elapsed time, average recognition rate, was proposed in [63]. A hybrid method for hand segmentation based on depth and color data acquired by the Kinect sensor with the help of skeletal data were proposed in [64]. In this method, the image threshold is applied to the depth frame and the super-pixel segmentation method is used to extract the hand from the color frame, then the two results are combined for robust segmentation. Figure 9 show an example on skeleton recognition.

According to information mentioned in Table 4. The depth camera provides good accuracy for segmentation, because not affected by lightening variations and cluttered background. However, the main issue is in the range of detection. The Kinect V1 sensor has an embedded system in which gives feedback information received by depth sensor as a metadata, which gives information about human body joint coordinate. The Kinect V1 provides information used to track skeletal joint up to 20 joints, that’s help to module the hand skeleton. While Kinect V2 sensor can tracking joint as 25 joints and up to six people at the same time with full joints tracking. With a range of detection between (0.5–4.5) meter.

#### 2.2.5. Depth-Based Recognition

Approaches have proposed for solving hand gesture recognition using different types of cameras. A depth camera provides 3D geometric information about the object [65]. Previously, both major approximations were utilized: TOF precepts and light coding. The 3D data from a depth camera directly reflects the depth field if compared with a color image which contains only a projection [66]. Using this approach, the lighting, shade, and color did not affect the result image. However, the cost, size and availability of the depth camera will limit its use [67]. Table 5 presents a set of research papers that use different segmentation techniques based on depth recognition to detect ROI.

**Figure 10:**
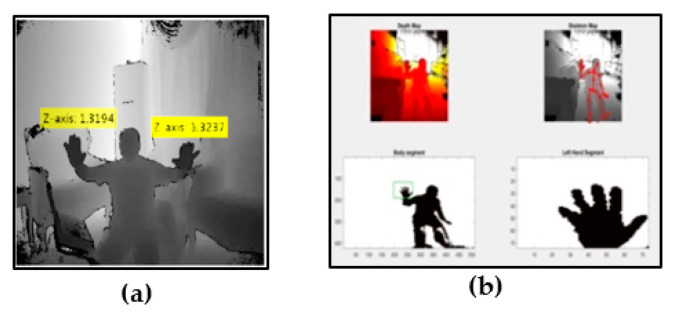
Depth-based recognition: (**a**) hand joint distance from camera; (**b**) different feature extraction using Kinect depth sensor.

The finger earth mover’s distance (FEMD) approach was evaluated in terms of speed and precision, and then compared with the shape-matching algorithm using the depth map and color image acquired by the Kinect camera [65]. Improved depth threshold segmentation was offered in [68], by combining depth and color information using the hierarchical scan method, then hand segmentation by the local neighbor method; this approach gives a result over a range of up to two meters. A new method was proposed in [69], based on a near depth range of less than 0.5 m where skeletal data were not provided by Kinect. This method was implemented using two image frames, depth and infrared. A depth threshold was used in order to segment the hand, then a K-mean algorithm was applied to obtain both user’s hand pixels [70]. Next, Graham’s scan algorithm was used to detect the convex hulls of the hand in order to merge with the result of the contour tracing algorithm to detect the fingertip. The depth image frame was analyzed to extract 3D hand gestures in real time, which were executed using frame differences to detect moving objects [71]. The foremost region was utilized and classified using an automatic state machine algorithm. The skin–motion detection technique was used to detect the hand, then Hu moments were applied to feature extraction, after which HMM was used for gesture recognition [72]. Depth range was utilized for hand segmentation, then Otsu’s method was used for applying threshold value to the color frame after it was converted into a gray frame [14]. A kNN classifier was then used to classify gestures. In [73], where the hand was segmented based on depth information using a distance method, background subtraction and iterative techniques were applied to remove the depth image shadow and decrease noise. In [74], the segmentation used 3D depth data selected using a threshold range. In [75], the proposed algorithm used an RGB color frame, which converted to a binary frame using Otsu’s global threshold. After that, a depth range was selected for hand segmentation and then the two methods were aligned. Finally, the kNN algorithm was used with Euclidian distance for finger classification. Depth data and an RGB frame were used together for robust hand segmentation and the segmented hand matched with the dataset classifier to identify the fingertip [76]. This framework was based on distance from the device and shape based matching. The fingertips selected using depth threshold and the K-curvature algorithm based on depth data were presented in [77]. A novel segmentation method was implemented in [78] by integrating RGB and depth data, and classification was offered using speeded up robust features (SURF). Depth information with skeletal and color data were used in [79], to detect the hand, then the segmented hand was matched with the dataset using SVM and artificial neural networks (ANN) for recognition. The authors concluded that ANN was more accurate than SVM. Figure 10 shows an example of segmentation using Kinect depth sensor.

#### 2.2.6. 3D Model-Based Recognition

The 3D model essentially depends on 3D Kinematic hand model which has a large degree of freedom, where hand parameter estimation obtained by comparing the input image with the two-dimensional appearance projected by three-dimensional hand model. In addition, the 3D model introduces human hand feature as pose estimation by forming volumetric or skeletal or 3D model that identical to the user’s hand. Where the 3D model parameter updated through the matching process. Where the depth parameter is added to the model to increase accuracy. Table 6 presents a set of research papers based on 3D model.

**Figure 11:**
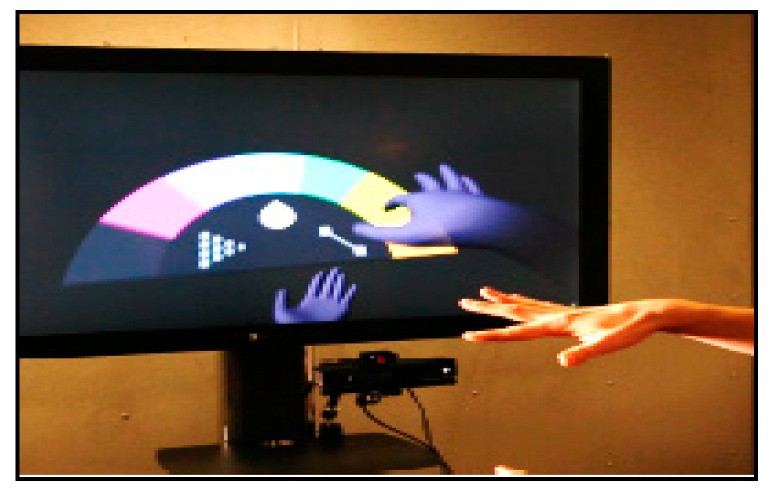
3D hand model interaction with virtual system [83].

A study by Tekin et al. [80] proposed a new model to understand interactions between 3D hands and object using single RGB image, where single image is trained end-to-end using neural network, and show jointly estimation of the hand and object poses in 3D. Wan et al. [81] proposed 3D hand pose estimation from single depth map using self-supervision neural network by approximating the hand surface with a set of spheres. A novel of estimating full 3D hand shape and pose presented by Ge et al. [82] based on single RGB image. Where Graph Convolutional Neural Network (Graph CNN) utilized to reconstruct full 3D mesh for hand surface. Another study by Taylor et al. [83] proposed a new system tracking human hand by combine surface model with new energy function which continuously optimized jointly over pose and correspondences, which can track the hand for several meter from the camera. Malik et al. [84] proposed a novel CNN-based algorithm which automatically learns in order to segment hand from a raw depth image and estimate 3D hand pose estimation including the structural constraints of hand skeleton. Tsoli et al. [85] presented a novel method to track a complex deformable object in interaction with a hand. Chen et al. [86] proposed self-organizing hand network SO—Hand Net—which achieved 3D hand pose estimation via semi-supervised learning. Where end-to-end regression method utilized for single depth image to estimation 3D hand pose. Another study by Ge et al. [87] proposed a point-to-point regression method for 3D hand pose estimation in single depth images. Wu et al. [88] proposed novel hand pose estimation from a single depth image by combine detection based method and regression-based method to improve accuracy. Cai et al. [89] present one-way to adapt a weakly labeled real-world dataset from a fully annotated synthetic dataset with the aid of low-cost depth images and take only RGB inputs for 3D joint predictions. Figure 11 shows an example of a 3D hand model interaction with virtual system.

There are some reported limitations, such as 3D hand required a large dataset of images to formulate the characteristic shapes of the hand in case multi-view. Moreover, the matching process considers time consumption, also computation costly and less ability to treat unclear views.

#### 2.2.7. Deep-Learning Based Recognition

The artificial intelligence offers a good and reliable technique used in a wide range of modern applications because of using a learning role principle. The deep learning used multilayers for learning data and gives a good prediction out result. The most challenges facing this technique is required dataset to learn algorithm which may affect time processing. Table 7 presents a set of research papers that use different techniques based on deep-learning recognition to detect ROI.

**Figure 12:**
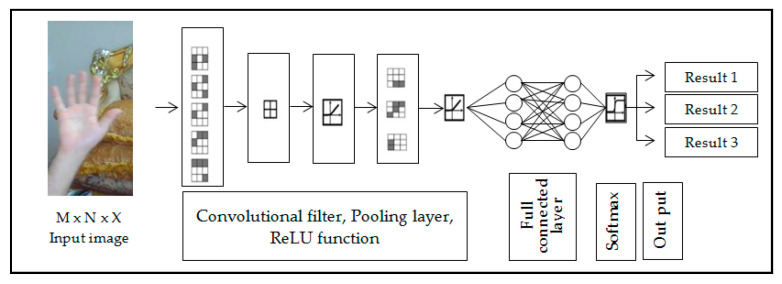
Simple example on deep learning convolutional neural network architecture.

Authors proposed seven popular hand gestures which captured by mobile camera and generate 24,698 image frames. The feature extraction and adapted deep convolutional neural network (ADCNN) utilized for hand classification. The experiment evaluates result for the training data 100% and testing data 99%, with execution time 15,598 s [90]. While other proposed systems used webcam in order to track hand. Then used skin color (Y–Cb–Cr color space) technique and morphology to remove the background. In addition, kernel correlation filters (KCF) used to track ROI. The resulted image enters into a deep convolutional neural network (CNN). Where the CNN model used to compare performance of two modified from Alex Net and VGG Net. The recognition rate for training data and testing data, respectively 99.90% and 95.61% in [91]. A new method based on deep convolutional neural network, where the resized image directly feds into the network ignoring segmentation and detection stages in orders to classify hand gestures directly. The system works in real time and gives a result with simple background 97.1% and with complex background 85.3% in [92]. The depth image produced by Kinect sensor used to segment color image then skin color modeling combined with convolution neural network, where error back propagation algorithm applied to modify the threshold and weights for the neural network. The SVM classification algorithm added to the network to enhance result in [93]. Other research study used Gaussian Mixture model (GMM) to filter out non-skin colors of an image which used to train the CNN in order to recognize seven hand gestures, where the average recognition rate 95.96 % in [94]. The next proposed system used long-term recurrent convolutional network-based action classifier, where multiple frames sampled from the video sequence recorded is fed to the network. In order to extract the representative frames, the semantic segmentation-based de-convolutional neural network is used. The tiled image patterns and tiled binary patterns are utilized to train the de-convolutional network in [95]. A double-channel convolutional neural network (DC-CNN) is proposed by [96] where the original image preprocessed to detect the edge of the hand before fed to the network. The each of two-channel CNN has a separate weight and softmax classifier used to classify output results. The proposed system gives recognition rate of 98.02%. Finally, a new neural network based on SPD manifold learning for skeleton-based hand gesture recognition proposed by [97]. Figure 12 below shown example on deep learn convolution neural network.

## 3. Application Areas of Hand Gesture Recognition Systems

**Figure 13:**
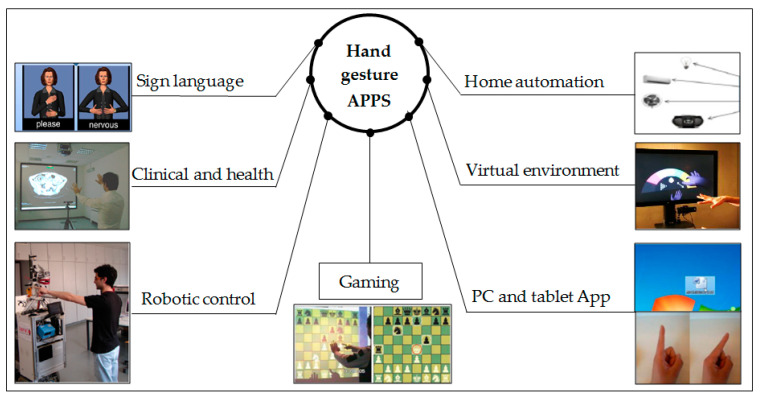
Most common application area of hand gesture interaction system (the image of Figure 13 is adapted from [12,14,42,76,83,98,99]).

Research into hand gestures has become an exciting and relevant field; it offers a means of natural interaction and reduces the cost of using sensors in terms of data gloves. Conventional interactive methods depend on different devices such as a mouse, keyboard, touch screen, joystick for gaming and consoles for machine controls. The following sections describe some popular applications of hand gestures. Figure 13 shows the most common application area deal with hand gesture recognition techniques.

### 3.1. Clinical and Health

During clinical operations, a surgeon may need details about the patient’s entire body structure or a detailed organ model in order to shorten the operating time or increase the accuracy of the result. This is achieved by using a medical imaging system such as MRI, CT or X-ray system [10,99], which collects data from the patient’s body and displays them on the screen as a detailed image. The surgeon can facilitate interaction with the viewed images by performing hand gestures in front of the camera using a computer vision technique. These gestures can enable some operations such as zooming, rotating, image cropping and going to the next or previous slide without using any peripheral device such as a mouse, keyboard or touch screen. Any additional equipment requires sterilization, which can be difficult in the case of keyboards and touch screen. In addition, hand gestures can be used for assistive purpose such as wheelchair control [43].

### 3.2. Sign Language Recognition

Sign language is an alternative method used by people who are unable to communicate with others by speech. It consists of a set of gestures wherein every gesture represents one letter, number or expression. Many research papers have proposed recognition of sign language for deaf-mute people, using a glove-attached sensor worn on the hand that gives responses according to hand movement. Alternatively, it may involve uncovered hand interaction with the camera, using computer vision techniques to identify the gesture. For both approaches mentioned above, the dataset used for classification of gestures matches a real-time gesture made by the user [11,42,50].

### 3.3. Robot Control

Robot technology is used in many application fields such as industry, assistive services [100], stores, sports and entertainment. Robotic control systems use machine learning techniques, artificial intelligence and complex algorithms to execute a specific task, which lets the robotic system, interact naturally with the environment and make an independent decision. Some research proposes computer vision technology with a robot to build assistive systems for elderly people. Other research uses computer vision to enable a robot to ask a human for a proper path inside a specific building [12].

### 3.4. Virtual Environment

Virtual environments are based on a 3D model that needs a 3D gesture recognition system in order to interact in real time as a HCI. These gestures may be used for modification and viewing or for recreational purposes, such as playing a virtual piano. The gesture recognition system utilizes a dataset to match it with an acquired gesture in real time [13,78,83].

### 3.5. Home Automation

Hand gestures can be used efficiently for home automation. Shaking a hand or performing some gesture can easily enable control of lighting, fans, television, radio, etc. They can be used to improve older people’s quality of life [14].

### 3.6. Personal Computer and Tablet

Hand gestures can be used as an alternative input device that enables interaction with a computer without a mouse or keyboard, such as dragging, dropping and moving files through the desktop environment, as well as cut and paste operations [19,69,76]. Moreover, they can be used to control slide show presentations [15]. In addition, they are used with a tablet to permit deaf-mute people to interact with other people by moving their hand in front of tablet’s camera. This requires the installation of an application that translates sign language to text, which is displayed on the screen. This is analogous to the conversion of acquired voice to text.

### 3.7. Gestures for Gaming

The best example of gesture interaction for gaming purposes is the Microsoft Kinect Xbox, which has a camera placed over the screen and connects with the Xbox device through the cable port. The user can interact with the game by using hand motions and body movements that are tracked by the Kinect camera sensor [16,98].

## 4. Research Gaps and Challenges

From the previous sections, it is easy to identify the research gap, since most research studies focus on computer applications, sign language and interaction with a 3D object through a virtual environment. However, many research papers deal with enhancing frameworks for hand gesture recognition or developing new algorithms rather than executing a practical application with regard to health care. The biggest challenge encountered by the researcher is in designing a robust framework that overcomes the most common issues with fewer limitations and gives an accurate and reliable result. Most proposed hand gesture systems can be divided into two categories of computer vision techniques. First, a simple approach is to use image processing techniques via Open-NI library or OpenCV library and possibly other tools to provide interaction in real time, which considers time consumption because of real-time processing. This has some limitations, such as background issues, illumination variation, distance limit and multi-object or multi-gesture problems. A second approach uses dataset gestures to match against the input gesture, where considerably more complex patterns require complex algorithm. Deep learning technique and artificial intelligence techniques to match the interaction gesture in real time with dataset gestures already containing specific postures or gestures. Although this approach can identify a large number of gestures, it has some drawbacks in some cases, such as missing some gestures because of the classification algorithms accuracy contrast. In addition, it takes time more than first approach because of the matching dataset in case of using a large number of the dataset. In addition, the dataset of gestures cannot be used by other frameworks.

## 5. Conclusions

Hand gesture recognition addresses a fault in interaction systems. Controlling things by hand is more natural, easier, more flexible and cheaper, and there is no need to fix problems caused by hardware devices, since none is required. From previous sections, it was clear to need to put much effort into developing reliable and robust algorithms with the help of using a camera sensor has a certain characteristic to encounter common issues and achieve a reliable result. Each technique mentioned above, however, has its advantages and disadvantages and may perform well in some challenges while being inferior in others.

## Figures and Tables

**Table 1:** Set of research papers that have used skin color detection for hand gesture and finger counting application.

Author	Type of Camera	Resolution	Techniques/Methods for Segmentation	Feature Extract Type	Classify Algorithm	Recognition Rate	No. of Gestures	Application Area	Invariant Factor	Distance from Camera
[37]	off-the-shelf HD webcam	16 Mp	Y–Cb–Cr	finger count	maximum distance of centroid two fingers	70% to 100%	14 gestures	HCI	light intensity, size, noise	150 to 200 mm
[38]	computer camera	320 × 250 pixels	Y–Cb–Cr	finger count	expert system	98%	6 gestures	deaf-mute people	heavy light during capturing	–
[11]	Fron-Tech E-cam (web camera)	10 Mp	RGB threshold & edge detection Sobel method	A–Z alphabet hand gesture	feature matching (Euclidian distance)	90.19%	26 static gestures	(ASL) American sign language	–	1000 mm
[15]	webcam	640 × 480 pixels	HIS & distance transform	finger count	distance transform method & circular profiling	100% > according limitation	6 gestures	control the slide during a presentation	location of hand	–
[39]	webcam	–	HIS & frame difference & Haar classifier	dynamic hand gestures	contour matching difference with the previous	–	hand segment	HCI	sensitive to moving background	–
[40]	webcam	640 × 480 pixels	HSV & motion detection (hybrid technique)	hand gestures	(SPM) classification technique	98.75%	hand segment	HCI	–	–
[41]	video camera	640 × 480 pixels	HSV & cross-correlation	hand gestures	Euclidian distance	82.67%	15 gestures	man–machine interface (MMI)	–	–
[42]	digital or cellphone camera	768 × 576 pixels	HSV	hand gestures	division by shape	–	hand segment	Malaysian sign language	objects have the same skin color some & hard edges	–
[43]	web camera	320 × 240 pixels	red channel threshold segmentation method	hand postures	combine information from multiple cures of the motion, color and shape	100%	5 hand postures	HCI wheelchair control	–	–
[44]	Logitech portable webcam C905	320 × 240 pixels	normalized R, G, original red	hand gestures	Haar-like directional patterns & motion history image	93.13 static 95.07 dynamic Percent	2 static 4 dynamic gestures	man–machine interface (MMI)	–	(< 1) mm (1000–1500) mm (1500–2000) mm
[45]	high resolution cameras	640 × 480 pixels	HIS & Gaussian mixture model (GMM) & second histogram	hand postures	Haarlet-based hand gesture	98.24% correct classification rate	10 postures	manipulating 3D objects & navigating through a 3D model	changes in illumination	–
[46]	ToF camera & AVT Marlin color camera	176 × 144 & 640 × 480 pixels	histogram-based skin color probability & depth threshold	hand gestures	2D Haarlets	99.54%	hand segment	real-time hand gesture interaction system	–	1000 mm

Table footer: –: none.

**Table 2:** A set of research papers that have used appearance-based detection for hand gesture application.

Author	Type of Camera	Resolution	Techniques/ Methods for Segmentation	Feature Extract Type	Classify Algorithm	RECOGNITION RATE	No. of Gestures	Application Area	Dataset Type	Invariant Factor	Distance from Camera
[49]	Logitech Quick Cam web camera	320 × 240 pixels	Haar -like features & AdaBoost learning algorithm	hand posture	parallel cascade structure	above 90%	4 hand postures	real-time vision-based hand gesture classification	Positive and negative hand sample collected by author	–	–
[50]	webcam-1.3	80 × 64 resize image for train	OTSU & canny edge detection technique for gray scale image	hand sign	feed-forward back propagation neural network	92.33%	26 static signs	American Sign Language	Dataset created by author	low differentiation	different distances
[51]	camera video	320 × 240 pixels	Gaussian model describes hand color in HSV & AdaBoost algorithm	hand gesture	palm–finger configuration	93%	6 hand gestures	real-time hand gesture recognition method	–	–	–
[52]	camera–projector system	384 × 288 pixels	background subtraction method	hand gesture	Fourier-based classification	87.7%	9 hand gestures	user-independent application	ground truth data set collected manually	point coordinates geometrically distorted & skin color	–
[53]	Monocular web camera	320 × 240 pixels	combine Y–Cb–Cr & edge extraction & parallel finger edge appearance	hand posture based on finger gesture	finger model	–	14 static gestures	substantial applications	The test data are collected from videos captured by web-camera	variation in lightness would result in edge extraction failure	≤ 500 mm

Table footer: –: none.

**Table 3:** A set of research papers that have used motion-based detection for hand gesture application.

Author	Type of Camera	Resolution	Techniques/ Methods for Segmentation	Feature Extract Type	Classify Algorithm	Recognition Rate	No. of Gestures	Application Area	Dataset Type	Invariant Factor	Distance from Camera
[54]	off-the-shelf cameras	–	RGB, HSV, Y–Cb–Cr & motion tracking	hand gesture	histogram distribution model	97.33%	10 gestures	human–computer interface	Data set created by author	other object moving and background issue	–
[55]	Canon GL2 camera	720 × 480 pixels	face detection & optical flow	motion gesture	leave-one-out cross-validation	–	7 gestures	gesture recognition system	Data set created by author	–	–
[56]	time of flight (TOF) SR4000	176 × 144 pixels	depth information, motion patterns	motion gesture	motion patterns compared	95%	26 gestures	interaction with virtual environments	cardinal directions dataset	depth range limitation	3000 mm
[57]	digital camera	–	YUV & CAMShift algorithm	hand gesture	naïve Bayes classifier	high	unlimited	human and machine system	Data set created by author	changed illumination, rotation problem, position problem	–

Table footer: –: none.

**Table 4:** Set of research papers that have used skeleton-based recognition for hand gesture application.

Author	Type of Camera	Resolution	Techniques/ Methods for Segmentation	Feature Extract Type	Classify Algorithm	Recognition Rate	No. of Gestures	Application Area	Dataset Type	Invariant Factor	Distance from Camera
[58]	Kinect camera depth sensor	512 × 424 pixels	Euclidean distance & geodesic distance	fingertip	skeleton pixels extracted	–	hand tracking	real time hand tracking method	–	–	–
[59]	Intel Real Sense depth camera	–	skeleton data	hand-skeletal joints’ positions	convolutional neural network (CNN)	91.28% 84.35%	14 gestures 28 gestures	classification method	Dynamic Hand Gesture-14/28 (DHG) dataset	only works on complete sequences	–
[60]	Kinect camera	240 × 320 pixels	Laplacian-based contraction	skeleton points clouds	Hungarian algorithm	80%	12 gestures	hand gesture recognition method	ChaLearn Gesture Dataset (CGD2011)	HGR less performance in the viewpoint 0◦condition	–
[61]	RGB video sequence recorded	–	vision-based approach & skeletal data	hand and body skeletal features	skeleton classification network	–	hand gesture	sign language recognition	LSA64 dataset	difficulties in extracting skeletal data because of occlusions	–
[62]	Intel Real Sense depth camera	640 × 480 pixels	depth and skeletal dataset	hand gesture	supervised learning classifier support vector machine (SVM) with a linear kernel	88.24% 81.90%	14 gestures 28 gestures	hand gesture application	Create SHREC 2017 track “3D Hand Skeletal Dataset	–	–
[63]	Kinect v2 camera sensor	512 × 424 pixels	depth metadata	dynamic hand gesture	SVM	95.42%	10 gesture 26 gesture	Arabic numbers (0–9) letters (26)	author own dataset	low recognition rate, “O”, “T” and “2”	–
[64]	Kinect RGB camera & depth sensor	640 × 480	skeleton data	hand blob	–	–	hand gesture	Malaysian sign language	–	–	–

Table footer: –: none.

**Table 5:** Set of research papers that have used depth-based detection for hand gesture and finger counting application.

Author	Type of Camera	Resolution	Techniques/ Methods for Segmentation	Feature Extract Type	Classify Algorithm	Recognition Rate	No. of Gestures	Application Area	Invariant Factor	Distance from Camera
[65]	Kinect V1	RGB - 640 × 480 depth - 320 × 240	threshold & near-convex shape	finger gesture	finger–earth movers distance (FEMD)	93.9%	10 gestures	human–computer interactions (HCI)	–	–
[68]	Kinect V2	RGB - 1920 × 1080 depth - 512 × 424	local neighbor method & threshold segmentation	fingertip	convex hull detection algorithm	96%	6 gestures	natural human–robot interaction	–	(500–2000) mm
[69]	Kinect V2	Infrared sensor depth - 512 × 424	operation of depth and infrared images	finger counting & hand gesture	number of separate areas	–	finger count & two hand gestures	mouse-movement controlling	–	< 500 mm
[70]	Kinect V1	RGB - 640 × 480 depth - 320 × 240	depth thresholds	finger gesture	finger counting classifier & finger name collect & vector matching	84% one hand 90% two hand	9 gestures	chatting with speech	–	(500–800) mm
[71]	Kinect V1	RGB - 640 × 480 depth - 320 × 240	frame difference algorithm	hand gesture	automatic state machine (ASM)	94%	hand gesture	human–computer interaction	–	–
[72]	Kinect V1	RGB - 640 × 480 depth - 320 × 240	skin & motion detection & Hu moments an orientation	hand gesture	discrete hidden Markov model (DHMM)	–	10 gestures	human–computer interfacing	–	–
[14]	Kinect V1	depth - 640 × 480	range of depth image	hand gestures 1–5	kNN classifier & Euclidian distance	88%	5 gestures	electronic home appliances	–	(250–650) mm
[73]	Kinect V1	depth - 640 × 480	distance method	hand gesture	–	–	hand gesture	human–computer interaction (HCI)	–	–
[74]	Kinect V1	depth - 640 × 480	threshold range	hand gesture	–	–	hand gesture	hand rehabilitation system	–	400–1500 mm
[75]	Kinect V2	RGB - 1920 × 1080 depth - 512 × 424	Otsu’s global threshold	finger gesture	kNN classifier & Euclidian distance	90%	finger count	human–computer interaction (HCI)	hand not identified if it’s not connected with boundary	250–650 mm
[76]	Kinect V1	RGB - 640 × 480 depth - 640 × 480	depth-based data and RGB data together	finger gesture	distance from the device and shape bases matching	91%	6 gesture	finger mouse interface	–	500––800 mm
[77]	Kinect V1	depth - 640 × 480	depth threshold and K-curvature	finger counting	depth threshold and K-curvature	73.7%	5 gestures	picture selection application	detection fingertips should though the hand was moving or rotating	–
[78]	Kinect V1	RGB - 640 × 480 depth - 320 × 240	integrate the RGB and depth information	hand gesture	forward recursion & SURF	90%	hand gesture	virtual environment	–	–
[79]	Kinect V2	depth - 512 × 424	skeletal data stream & depth & color data streams	hand gesture	support vector machine (SVM) & artificial neural networks (ANN)	93.4% for SVM 98.2% for ANN	24 alphabets hand gesture	American Sign Language	–	500––800 mm

Table footer: –: none.

**Table 6:** Set of research papers that have used 3D model-based recognition for HCI, VR and human behavior application.

Author	Type of Camera	Techniques/ Methods for Segmentation	Feature Extract Type	Classify Algorithm	Type of Error	Hardware Run	Application Area	Dataset Type	Runtime Speed
[80]	RGB camera	network directly predicts the control points in 3D	3D hand poses, 6D object poses ,object classes and action categories	PnP algorithm & Single-shot neural network	Fingertips 48.4 mm Object coordinates 23.7 mm	real-time speed of 25 fps on an NVIDIA Tesla M40	framework for understanding human behavior through 3Dhand and object interactions	First-person hand action (FPHA) dataset	25 fps
[81]	Prime sense depth cameras	depth maps	3D hand pose estimation & sphere model renderings	Pose estimation neural network	mean joint error (stack = 1) 12.6 mm (stack = 2) 12.3 mm	–	design hand pose estimation using self-supervision method	NYU Hand Pose Dataset	–
[82]	RGB-D camera	Single RGB image direct feed to the network	3D hand shape and pose	train networks with full supervision	Mesh error 7.95 mm Pose error 8.03 mm	Nvidia GTX 1080 GPU	design model for estimate 3D hand shape from a monocular RGB image	Stereo hand pose tracking benchmark (STB) & Rendered Hand Pose Dataset (RHD)	50 fps
[83]	Kinect V2 camera	segmentation mask Kinect body tracker	hand	machine learning	Marker error 5% subset of the frames in each sequence & pixel classification error	CPU only	interactions with virtual and augmented worlds	Finger paint dataset & NYU dataset used for comparison	high frame-rate
[84]	raw depth image	CNN-based hand segmentation	3D hand pose regression pipeline	CNN-based algorithm	3D Joint Location Error 12.9 mm	Nvidia Geforce GTX 1080 Ti GPU	applications of virtual reality (VR)	dataset contains 8000 original depth images created by authors	–
[85]	Kinect V2 camera	bounding box around the hand & hand mask	hand	appearance and the kinematics of the hand	percentage of template vertices over all frames	–	Interaction with deformable object & tracking	synthetic dataset generated with the Blender modeling software	–
[86]	RGBD data from 3 Kinect devices	regression-based method & hierarchical feature extraction	3D hand pose estimation	3D hand pose estimation via semi-supervised learning.	Mean error 7.7 mm	NVIDIA TITAN Xp GPU	human–computer interaction (HCI), computer graphics and virtual/augmented reality	For evaluation ICVL Dataset & MSRA Dataset & NYU Dataset	58 fps
[87]	single depth images.	depth image	3D hand pose	3D point cloud of hand as network input and outputs heat-maps	mean error distances	Nvidia TITAN Xp GPU	(HCI), computer graphics and virtual/augmented reality	For evaluation NYU dataset & ICVL dataset & MSRA datasets	41.8 fps
[88]	depth images	predicting heat maps of hand joints in detection-based methods	hand pose estimation	dense feature maps through intermediate supervision in a regression-based framework	mean error 6.68 mm maximal per-joint error 8.73 mm	GeForce GTX 1080 Ti	(HCI), virtual and mixed reality	For evaluation ‘HANDS 2017′ challenge dataset & first-person hand action	–
[89]	RGB-D cameras	–	3D hand pose estimation	weakly supervised method	mean error 0.6 mm	GeForce GTX 1080 GPU with CUDA 8.0.	(HCI), virtual and mixed reality	Rendered hand pose (RHD) dataset	–

Table footer: –: none.

**Table 7:** Set of research papers that have used deep-learning-based recognition for hand gesture application.

Author	Type of Camera	Resolution	Techniques/ Methods for Segmentation	Feature Extract Type	Classify Algorithm	Recognition Rate	No. of Gestures	Application Area	Dataset Type	Hardware Run
[90]	Different mobile cameras	HD and 4k	features extraction by CNN	hand gestures	Adapted Deep Convolutional Neural Network (ADCNN)	training set 100% test set 99%	7 hand gestures	(HCI) communicate for people was injured Stroke	Created by video frame recorded	Core™ i7-6700 CPU @ 3.40 GHz
[91]	webcam	–	skin color detection and morphology & background subtraction	hand gestures	deep convolutional neural network (CNN)	training set 99.9% test set 95.61%	6 hand gestures	Home appliance control (smart homes)	4800 image collect for train and 300 for test	–
[92]	RGB image	640 × 480 pixels	No segment stage Image direct fed to CNN after resizing	hand gestures	deep convolutional neural network	simple backgrounds 97.1% complex background 85.3%	7 hand gestures	Command consumer electronics device such as mobiles phones and TVs	Mantecón et al.* dataset for direct testing	GPU with 1664 cores, base clock of 1050 MHz
[93]	Kinect	–	skin color modeling combined with convolution neural network image feature	hand gestures	convolution neural network & support vector machine	98.52%	8 hand gestures	–	image information collected by Kinect	CPUE 5-1620v4, 3.50 GHz
[94]	Kinect	Image size 200 × 200	skin color -Y–Cb–Cr color space & Gaussian Mixture model	hand gestures	convolution neural network	Average 95.96%	7 hand gestures	human hand gesture recognition system	image information collected by Kinect	–
[95]	video sequences recorded	–	Semantic segmentation based deconvolution neural network	hand gesture motion	convolution network (LRCN) deep	95%	9 hand gestures	intelligent vehicle applications	Cambridge gesture recognition dataset	Nvidia Geforce GTX 980 graphics
[96]	image	Original images in the database 248 × 256 or 128 × 128 pixels	Canny operator edge detection	hand gesture	double channel convolutional neural network (DC-CNN) & softmax classifier	98.02%	10 hand gestures	man–machine interaction	Jochen Triesch Database (JTD) & NAO Camera hand posture Database (NCD)	Core i5 processor
[97]	Kinect	–	–	Skeleton-based hand gesture recognition.	neural network based on SPD	85.39%	14 hand gestures	–	Dynamic Hand Gesture (DHG) dataset & First-Person Hand Action (FPHA) dataset	non-optimized CPU 3.4 GHz

Table footer: –: none.
